# The feasibility of task-sharing the identification, emergency treatment, and referral for women with pre-eclampsia by community health workers in India

**DOI:** 10.1186/s12978-018-0532-5

**Published:** 2018-06-22

**Authors:** Umesh Charanthimath, Marianne Vidler, Geetanjali Katageri, Umesh Ramadurg, Chandrashekhar Karadiguddi, Avinash Kavi, Anjali Joshi, Geetanjali Mungarwadi, Sheshidhar Bannale, Sangamesh Rakaraddi, Diane Sawchuck, Rahat Qureshi, Sumedha Sharma, Beth A. Payne, Peter von Dadelszen, Richard Derman, Laura A. Magee, Shivaprasad Goudar, Ashalata Mallapur, Mrutyunjaya Bellad, and the Community Level Interventions for Pre-eclampsia (CLIP) India Feasibility Working Group, Zulfiqar Bhutta, Sheela Naik, Anis Mulla, Namdev Kamle, Vaibhav Dhamanekar, Sharla K. Drebit, Chirag Kariya, Tang Lee, Jing Li, Mansun Lui, Asif R. Khowaja, Domena K. Tu, Amit Revankar

**Affiliations:** 1 KLE Academy of Higher Education and Research’s, J N Medical College, Belagavi, Karnataka India; 2 Department of Obstetrics and Gynaecology, and the Child and Family Research Unit, University of British, Columbia, Vancouver, BC Canada; 3 Department of Obstetrics and Gynaecology, S Nijalingappa Medical College, Bagalkot, Karnataka India; 4 Department of Community Medicine, S Nijalingappa Medical College, Bagalkot, Karnataka India; 5 Department of Pharmacology, S Nijalingappa Medical College, Bagalkot, Karnataka India; 6 Department of Anatomy, S Nijalingappa Medical College, Bagalkot, Karnataka India; 7 Department of Research, Vancouver Island Health Authority, Victoria, BC Canada; 8 Division of Women and Child Health, Aga Khan University, Karachi, Sindh Pakistan; 9 School of Life Course Sciences, Faculty of Life Sciences and Medicine, King’s College London, London, England; 10 Department Kings of Obstetrics, Thomas Jefferson University, Philadelphia, PA USA

**Keywords:** Task-sharing, Community health workers, Pre-eclampsia, Blood pressure, Antihypertensives, Magnesium sulphate

## Abstract

**Background:**

Hypertensive disorders are the second highest direct obstetric cause of maternal death after haemorrhage, accounting for 14% of maternal deaths globally. Pregnancy hypertension contributes to maternal deaths, particularly in low- and middle-income countries, due to a scarcity of doctors providing evidence-based emergency obstetric care. Task-sharing some obstetric responsibilities may help to reduce the mortality rates. This study was conducted to assess acceptability by the community and other healthcare providers, for task-sharing by community health workers (CHW) in the identification and initial care in hypertensive disorders in pregnancy.

**Methods:**

This study was conducted in two districts of Karnataka state in south India. A total of 14 focus group discussions were convened with various community representatives: women of reproductive age (*N* = 6), male decision-makers (*N* = 2), female decision-makers (*N* = 3), and community leaders (*N* = 3). One-to-one interviews were held with medical officers (*N* = 2), private healthcare OBGYN specialists (*N* = 2), senior health administrators (*N* = 2), Taluka (county) health officers (*N* = 2), and obstetricians (*N* = 4). All data collection was facilitated by local researchers familiar with the setting and language. Data were subsequently transcribed, translated and analysed thematically using NVivo 10 software.

**Results:**

There was strong community support for home visits by CHW to measure the blood pressure of pregnant women; however, respondents were concerned about their knowledge, training and effectiveness. The treatment with oral antihypertensive agents and magnesium sulphate in emergencies was accepted by community representatives but medical practitioners and health administrators had reservations, and insisted on emergency transport to a higher facility. The most important barriers for task-sharing were concerns regarding insufficient training, limited availability of medications, the questionable validity of blood pressure devices, and the ability of CHW to correctly diagnose and intervene in cases of hypertensive disorders of pregnancy.

**Conclusion:**

Task-sharing to community-based health workers has potential to facilitate early diagnosis of the hypertensive disorders of pregnancy and assist in the provision of emergency care. We identified some facilitators and barriers for successful task-sharing of emergency obstetric care aimed at reducing mortality and morbidity due to hypertensive disorders of pregnancy.

## Background

Globally, maternal mortality has fallen by 45% over the past two decades [1]. Since 1990, India has made significant progress in reducing the maternal mortality ratio by 68.7%. The latest estimate calculated by the World Bank was 174 per 100,000 live births. Furthermore, there has been a substantial increase in accessing antenatal care and delivery in hospital [2, 3]. Nevertheless, India accounts for 15% of global maternal deaths annually [4].

Hypertensive disorders of pregnancy (HDP) are some of the main causes of maternal death globally, and, in India, are estimated to cause 7.1% of maternal mortality. Symptomatic HDP, including pre-eclampsia, gestational hypertension and chronic hypertension, often occur late in pregnancy. Repeated blood pressure (BP) monitoring is advised in pregnancy for early detection of hypertension which would optimise outcomes [5]. Women with HDP require enhanced surveillance by appropriately trained healthcare professionals providing evidence-based care (including clinical, laboratory and ultrasound assessments) to guide timing of delivery along with initiation of life-saving therapies (use of oral antihypertensive and magnesium sulphate).

Targets for many health indicators are not met as expected and hence it is important to explore means of strengthening the health system. One of the barriers to providing universal coverage of health services is the inadequacies of health care professionals, especially in many low and middle Income countries (LMICs) [6]. In 2005, the World Health Organization (WHO) estimated that more than 90% percent of maternal deaths are avoidable with moderate levels of health care and task-sharing or shifting. Task-shifting, as defined by the WHO, is when “specific tasks are moved, where appropriate, from highly qualified health workers to health workers with shorter training and fewer qualifications in order to make more efficient use of available resources for health”. Task-sharing is a strategy in which health care workers take on additional duties with sufficient training and supervision [7]. Sharing of tasks from health professionals to community health workers (CHW) can improve access to care and optimize the use of limited human resources in many resource-poor settings [8]. “Task-sharing” the serial measurement of blood pressure, risk stratification, and initiation of both life-saving therapies and referral, may be effective in reducing HDP associated morbidity and mortality and serve to bridge the gap of health service delivery to women residing in rural India. This study aims to better understand the facilitators and barriers to implementation of this strategy in Karnataka, India.

## Study area

**Fig. 1:**
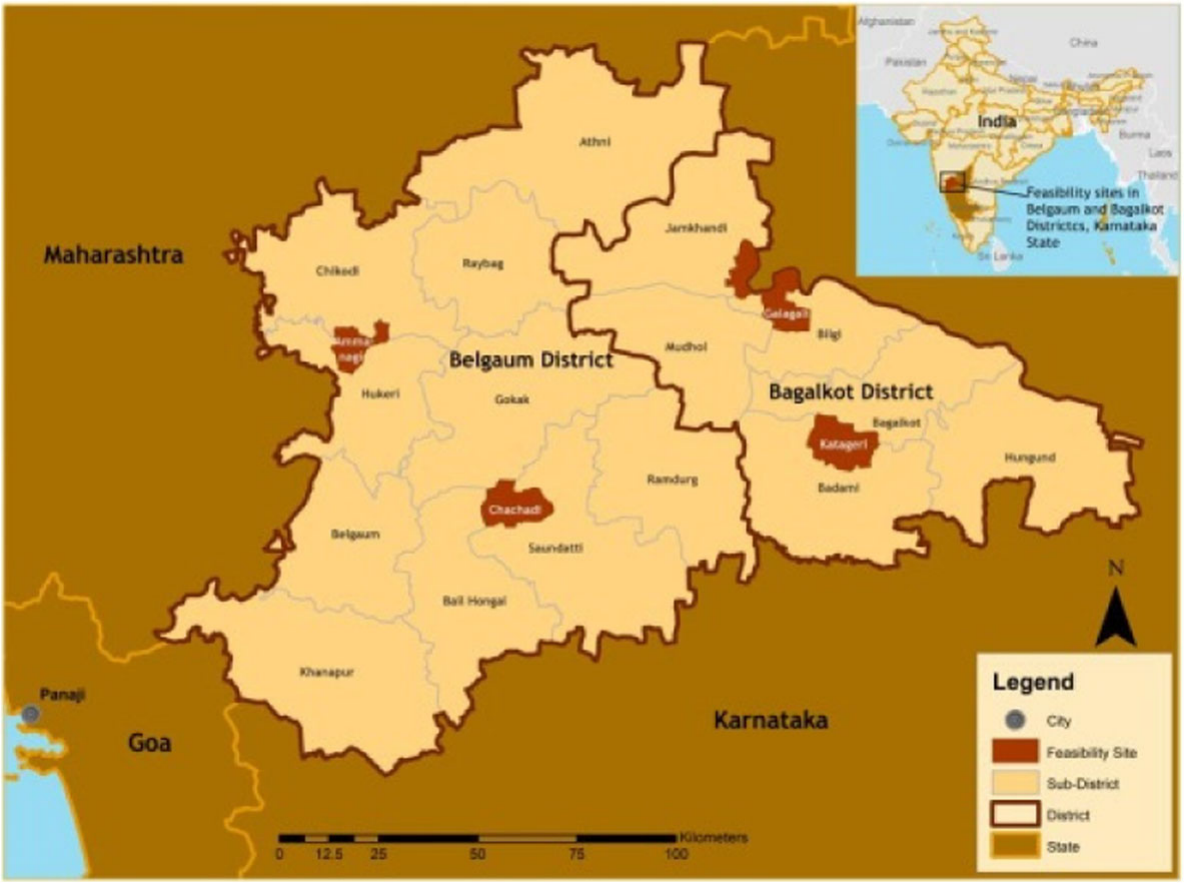
Map of the study site

This study was conducted in Belagavi and Bagalkote districts of rural Karnataka, in South India (Fig. 1). The healthcare infrastructures of Karnataka are inadequate for serving the rural and remote areas where there are health worker shortages and large distances from health facilities [3]. The National Rural Health Mission (NRHM) has instituted community health worker programmes throughout the region to increase service utilisation in the hope of improving health outcomes [3]. For study site characteristics, see Table 1.

**Table 1:** Site characteristics

	India	South India	Karnataka
Site characteristics			
Population	1028,610,328^a^		61095297^d^
# States	35	5	(30 Districts)
Dominant religion	Hindu^c^	Hindu^c^	Hindu^c^
Women’s literacy	55%^c^	68%^c^	58%^c^
Employment	36% currently employed^c^	41% currently employed^c^	40% currently employed^c^
Rural /Urban	32% urban^a^		39% urban^d^
Fertility rate	2.8^c^	1.9^c^	2.1^c^
Maternal mortality ratio	178 per 100,000 live births^b^	105 per 100,000 live births^a^	144 per 100,000 live births^a^
Maternal health care utilization			
ANY ANC	76.4%^c^	94%^c^	89%^c^
≥ 4 ANC	48%^c^	89%^c^ *(3+)*	76%^c^*(3+)*
Facility delivery (%)	39%^c^	79%^c^	65%^c^
Skilled attendant at delivery	47%^c^	84%^c^	70%^c^

^a^ *World Health Organization Country Profile: India 2012*

^b^ *Office of the Registrar of India, 2013*

^c^ *Demographic Health Survey 2013*

^d^ *Rural Health Statistics in India 2012*

## Community health workers

Community health workers in India include Auxiliary Nurse Midwives (ANM) and Accredited Social Health Activists (ASHA). ANMs provide care at the sub-centre, covering a population of 3000 to 5000 [9]. ANMs are trained in various topics related to maternal and child health, such as the provision of antenatal care, skilled attendance at delivery, postpartum care, and the management of pregnancy complications. NRHM guidelines from 2012 are used to train and authorise ANMs to administer 50% magnesium sulphate for cases of severe pre-eclampsia and eclampsia [10]. In 2005, the Government of India launched the ASHA programme to bring door-to-door health services to rural areas. ASHAs are females between the ages of 25 to 45 years, with an education equivalent to grade eight or higher, who are selected by the local government to serve in their residential areas. Each ASHA extends her services to a population of about 1000 individuals. ASHAs are trained to provide health care advice in the homes (provision of basic maternity care, child care and nutrition counselling), create community health awareness, conducting social mobilisation, treatment of infections, maintenance of health records and increase utilization of existing health services. ASHAs are trained in district training centres for a period of 4 weeks with the upgrading of knowledge being done by the Lady Health Visitors and Medical Officers of the respective primary health centres. ASHAs have been deemed to bridge the gap between the community, ANMs, primary health centres, and referral facilities [11].

## Methods

This qualitative study was conducted as part of a larger country assessment in the preparation of a cluster randomized control trial, the Community Level Interventions for Pre-eclampsia (CLIP) Trial (NCT01911494).

This study consisted of focus group discussions (FGD) and in-depth interviews (IDI). FGDs were preferred to encourage group dialogue from all participants. Researchers selected various participant groups including community leaders, male and female decision-makers and women of reproductive age. The community leaders are representatives from the local government or other members who are held in high esteem by the community. They play an important role in decision-making, affecting their respective locality. The male and female decision-makers were the family members who took responsibility for healthcare related decisions for the family. In-depth interviews were conducted with medical officers, senior health administrators and obstetricians from both private and government institutions. These groups were chosen to represent the spectrum of the communities’ and health care providers’ views (Table 2). Fourteen FGDs and twelve IDIs were conducted between January and March 2013. Data saturation was noted after these interviews and FGDs.

**Table 2:** Characteristics of focus group participants

#	Stakeholder Group	District	Education status				Total Participants
			A	B	C	D	
1	Community Leaders	Bagalkote	0	6	1	0	7
2	Community Leaders	Bagalkote	1	8	1	0	10
3	Community Leaders	Belagavi	–	–	–	10	10
4	Male Decision-Makers	Bagalkote	3	3	2	0	8
5	Male Decision-Makers	Belagavi	0	9	2	0	11
6	Female Decision-Makers	Bagalkote	8	2	0	0	10
7	Female Decision-Makers	Belagavi	12	4	2	0	18
8	Female Decision-Makers	Belagavi	11	2	0	0	13
9	Women of Reproductive Age	Belagavi	–	–	–	55	55
10	Women of Reproductive Age	Bagalkote	0	15	1	0	16
11	Women of Reproductive Age	Bagalkote	3	8	3	0	14
12	Women of Reproductive Age	Belagavi	0	15	2	0	17
13	Women of Reproductive Age	Belagavi	3	8	3	0	14
14	Women of Reproductive Age	Belagavi	0	10	6	0	16
			41 (18.7%)	90 (41.1%)	23 (10.5%)	65 (29.68%)	
**TOTAL**							219

A – Illiterate

B- Primary/Secondary Schooling

C – Pre university / University

D – Don’t Know

Local clinicians and researchers with the knowledge of cultural nuances and dynamics, with no known association with the respondents, were chosen and trained for qualitative study methods to facilitate interviews and focus group discussions. Focus group and interview guides were developed for the study and were semi-structured to promote a natural discussion progression. The FGDs were conducted in the local language, *Kannada*, to best promote interaction with participants and obtain the richest data. All FGDs were facilitated by one researcher and assisted by a second researcher who recorded field notes including non-verbal communication. IDIs were conducted in English. All FGDs and IDIs were audio recorded. The first FGD incorporating 55 women of reproductive age was conducted as a pilot to sensitise the local research team regarding effective ways of data collection in qualitative research.

The remaining stakeholder groups were convened separately and comprised a varied number of participants (between seven and eighteen). Participants were identified through local health system networks of ASHA and ANMs. Male and female decision-makers in the family were approached for participation when they accompanied women of reproductive age to local health centres.

All audio recordings were later transcribed verbatim and translated into English for analysis, with the incorporation of field notes. Data were analysed using NVivo 10 software. Transcripts were coded by one rater (MV), after which all coded transcripts and themes were cross-checked by the local research team to resolve or clarify any misinterpretation. Using deductive reasoning, results were then grouped into predetermined key themes. During analysis, inductive reasoning was used to incorporate new and unexpected ideas. This produced a comprehensive analysis structure to reflect the richness and variety of responses.

## Results

Participants in the community leaders FGD had diverse backgrounds: some were illiterate with no formal schooling while others had completed university education or college. They ranged in age between 24 and 51 years. Male decision-makers were aged between 18 to 57 years; 85% were illiterate, most identified themselves as labourers and farmers and were often the husband or father/father-in-law of the pregnant woman. Female decision-makers were 28 to 65 years of age, most had no formal schooling and could not read or write (71%), and the majority identified themselves as housewives and mothers-in-law. Women of reproductive age had an average age of 23 years, nearly all of them were housewives (91%), most were pregnant at the time of data collection (79%), and over half (66%) had at least one child under the age of five.

Two IDIs were conducted with medical officers, each responsible for one primary health centre which serves as the entry point to the health system. Senior district health system administrators (*N* = 4) were invited to participate, as they coordinate reproductive health services throughout the district and have a unique perspective. They are actively involved in the implementation of reproductive and child health services by local, state and central government. Three were obstetricians and gynaecologists; and one, a general surgeon.

Two obstetricians from secondary and four from tertiary facilities serving in the private and government sectors, with varying experience and training were interviewed. They provided specialist care to women coming from the study area. For more participant details, see Table 2 and Table 3.

**Table 3:** Characteristics of In-depth Interview participants

#	Stakeholder	Training	Level of Care	Pregnancies/ Week
1	Medical Officer	MBBS	Primary	50–60
2	Medical Officer	MBBS	Primary	10–15
3	Private Practitioner	MBBS, MD in OBG	Tertiary	40–50
4	Private Practitioner	MBBS, MD in OBG	Tertiary	280–300
5	Senior Health Administrator	MS General Surgery	*NA*	*NA*
6	Senior Health Administrator	MBBS & Diploma in OBG	*NA*	*NA*
7	Taluka Health Officer	MBBS, Diploma in OBG	Secondary	200–250
8	Taluka Health Officer	MBBS & Diploma OBG	Secondary	50–60
9	Obstetrician	MBBS, MD in OBG	Tertiary	250
10	Obstetrician	MBBS, DGO, MD in OBG	Tertiary	200–300
11	Obstetrician	MBBS, Diploma in OBG	Tertiary	45
12	Obstetrician	MBBS, MD in OBG	Tertiary	–

### Home blood pressure monitoring visits by ASHA workers

Most community leaders agreed that ASHAs could safely measure BP in the home; however, some had concerns regarding their knowledge, education, effectiveness, training, experience and supervision. Overall, there was an acceptance of ASHAs being tasked with the measurement of BP at home once they were appropriately trained.

*“In the future, ASHAs might do if they are well educated and trained about BP measurement” (Community Leader)*

*“A life is dependent on appropriate BP measurement. How will you come to know that ASHAs have understood? It is not possible. Is there any exam?” (Community Leader)*

Nearly all decision-makers agreed that a shift of this activity to ASHAs would be acceptable in their communities. In contrast, one group of male decision-makers strongly opposed this suggestion and argued that they would be unable to judge if an ASHA had not been properly trained and the possibility of a misdiagnosis was high.

*“Because even a MBBS qualified person who has studied for 4-5 years cannot do it, then how can an SSLC graduate do...if training would suffice then everybody would have taken training” (Male Decision-Maker)*

Two Medical officers and three obstetricians expressed confidence in ASHAs measuring BP if they were provided with a reliable easy-to-use digital device. Some obstetricians who were already using a digital apparatus spoke about the problems associated with these devices, such as erroneous readings, battery life and expressed concern whether ASHAs would be able to overcome these challenges. These practitioners did not comment on the technique required to measure accurate BP. They appeared to feel strongly that this job requires experience and qualification; and less-educated or under-trained workers like ASHAs could not do it effectively.

Most Women of reproductive age were happy to accept ASHAs for recording blood pressure at home. Health administrators did not support BP measurement by ASHAs. Private practitioners were not familiar with the capabilities of the ASHA workers and had no opinion or suggestions to make.

### Initiation of treatment with oral antihypertensive by ANMs

All community leaders and male decision maker supported treatment by ANMs except two, who strongly opposed treatment. The remaining participants supported this task-sharing by ANMs, but stressed the need for swift transport to the higher facility for further management. These respondents believed that ANMs had been adequately trained to provide oral emergency treatment but there should be someone who takes the responsibility for training and supervision.

***“*** *In my opinion, not all ANMs are experienced especially newly appointed ones. Some ANMs are experienced, such ANMs should be designated to give the medicines when the doctor is not there”. (Community Leader)*

*“No……No…. Who will do that? They cannot give treatment in an emergency”*

*(Male Decision-Maker)*

The women of reproductive age were ready to accept medications from ANMs in emergency situations. Some obstetricians accepted the new role for the ANM; however, some voiced concerns regarding their ability to manage side-effects resulting from the use of the medication. All obstetricians emphasised the importance of proper training and urgent referral.

*“I don’t support this. Methyldopa causes sedation so before giving that drug we convince them, we counsel them. So the same thing is done by ANM they will have to be well educated of everything” (OBGYN tertiary care hospital)*

### Administration of magnesium sulphate loading dose by ANMs

Community leaders accepted that with proper training, ANMs could be permitted to administer magnesium sulphate treatment in urgent cases. They expressed that such an emergency intervention might increase the likelihood of a woman’s survival before she reaches a referral centre. Female decision-makers and women of reproductive age expressed strong acceptance for receiving treatment from ANMs and expressed faith in care by ANMs. Obstetricians supported the administration of a loading dose of magnesium sulphate as it is safe intervention, needs no extensive monitoring and believed that it would reduce the complications associated with preeclampsia and eclampsia. All groups were in favour of giving magnesium sulphate but stressed appropriate training. A few mentioned that ANMs should contact a medical officer before giving the treatment and arrange for transport. Health administrators emphasised the importance of ensuring uninterrupted availability of drugs and proper training prior to task-sharing.

### Availability of transport and acceptability of referral

FGD and interview participants emphasized that treatment should not be continued at the community level and woman needed to be referred from the community to higher referral centre. With the availability of robust free ambulance services by the state government in this locality (free ambulance service on dialling 108), transport was not seen as much of a challenge. Women of reproductive age claimed that they would refuse emergency treatment and referral if a family member was unavailable to decide on her behalf or accompany her.

## Discussion

**Fig. 2:**
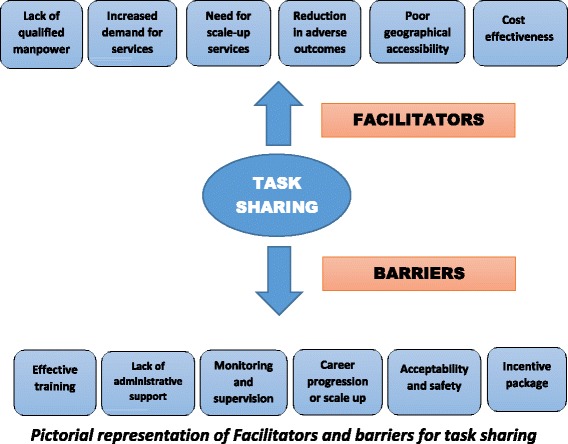
Pictorial representations of facilitators and barriers for task-sharing

The study aimed to identify facilitators and barriers for task-sharing in the identification and emergency treatment of pregnant women with HDP (Fig. 2). Enhanced antenatal and postnatal surveillance at home by the ASHA workers with measurement of BP and when indicated, administration of oral antihypertensives and intramuscular magnesium sulphate by the ANM prior to referral form the key components of the CLIP (NCT01911494) Trial.

There was strong community support for home visits by CHW to measure the blood pressure of pregnant women; however, respondents were concerned about their knowledge, training and effectiveness. The treatment with oral antihypertensive agents and magnesium sulphate in emergencies was accepted by community representatives but medical practitioners and health administrators had reservations, and insisted on emergency transport to a higher facility. The most important barriers for task-sharing were concerns regarding insufficient training, limited availability of medications, the questionable validity of blood pressure devices, and the ability of CHW to correctly diagnose and intervene in cases of hypertensive disorders of pregnancy.

Task-sharing is a process which has been proposed to overcome manpower shortages faced by the health care system. This shifts the tasks to “lay workers” or CHW who are not formally trained in the task assigned to them but can be expected to carry out the responsibilities after some training and with supervision [8]. This causes an improvement in early diagnosis and treatment of emergency conditions and is cost-effective [12, 13]. Task-sharing could improve access to health workers in resource-constrained settings and possibly have an impact on reducing mortality and morbidity [8].

More commonly, tasks expected from the “lay workers” are related to preventive and health promotional activities. The importance of “Community Embeddedness” of the lay worker has been stressed [14]. Women of reproductive age and decision makers in this study favoured home visits by the ASHA for BP monitoring. This has also been noted by other studies that found that this could be because it would reduce visits to the hospital for measurement of BP which were missed at times due to lack of knowledge, poor transportation facilities, competing responsibilities and commitments, loss of daily wages due to the visit and financial constraints [15]. People tend to preferably seek health care if the facility is close by rather than at a great distance [16]. Pregnant women and female decision-makers also supported this as the ASHA belongs to their locality and lives in close proximity; and could be helpful during emergencies, as other studies have reported [12].

The ASHA workers are not trained in measuring BP or other clinical examinations but are capable of identifying the danger signs of HDP [11]. The use of CHW for the measurement of BP in chronic hypertension has been reported in several studies, with favourable outcomes in the participants [17, 18]. A study done in north India demonstrated that ASHA workers could be trained to measure BP and concluded that they could be used for monitoring hypertension antenatally [19].

Some obstetricians raised concern regarding the use of an oscillometric digital apparatus, as well as validation and errors in recording. It is however important that the BP devices be validated for the purpose they are intended. The Microlife 3AS 1–2 semi-automatic handheld BP device, used by the ASHAs in the CLIP Trial has been validated for use in pregnant women including those with pre-eclampsia. This handheld device can be used effectively by unskilled personnel after minimal training [20, 21]. The concerns about errors in the BP readings can be substantially reduced by proper ongoing training sessions, validation and calibration of the device [22, 23].

WHO recognises that lay workers are more likely to be motivated if their tasks include curative aspects along with the preventive [8]. The ANMs were expected to administer methyldopa orally if the woman was found to have severe hypertension, in addition to magnesium sulphate injection intramuscularly in certain clinical scenarios, prior to referral. The ANMs are familiar with many drugs, Intravenous fluids, and injectable medications and vaccines [10]. Women of reproductive age and decision makers voiced trust in the ANM for treating women with HDP using oral antihypertensives and referring them to higher centres for further management. Since the antihypertensive in question here is in tablet form, skill development is not needed for administration.

Task-sharing in the provision of healthcare is not a new concept and has been implemented widely for the management of various conditions such as malaria, HIV, and tuberculosis (TB) [24–28]. Lewin et al. found that the use of lay health workers improved the uptake of immunization and breastfeeding practices; and also decreased morbidity and mortality from common childhood illnesses and additionally led to improved TB treatment outcomes [29].

Some obstetricians expressed concern about the ANM giving methyldopa due to concerns of adverse effects; however, it is widely recommended for use in HDP [30]. Methyldopa is found to be safe in HDP when used to prevent maternal and foetal complications. Though it may sometimes cause sedation or drowsiness, there is no evidence of acute complications in HDP with a loading dose of methyldopa [31–33].

The other task expected of the ANMs was the administration of magnesium sulphate injection intramuscularly before referral. The Magpie trial revealed that administration of magnesium sulphate leads to a 58% reduction in the risk of convulsions and its safety is well established [34, 35]. Magnesium sulphate is on the WHO Essential Medicines List for the use in severe pre-eclampsia and eclampsia [36]. Obstetricians interviewed in this study were also supportive of the administration of magnesium sulphate before referral and perceived it be safe. According to the guidelines for skilled birth attendants in India, ANMs are authorised to use 10 g magnesium sulphate as a deep intramuscular injection (5 g on each buttock) prior to referral in severe preeclampsia and eclampsia. Although provision of magnesium sulphate is in the current ANM guidelines, it is rarely followed in practice. This could be overcome by administrative support and effective training [10].

An adjunctive study conducted in tandem with the present study found that ANMs perceived magnesium sulphate to be an antihypertensive and nifedipine to be an anticonvulsant. It is necessary to address these misconceptions. Nevertheless, ANMs expressed confidence in the administration of intramuscular injections and this self-assessed competence could be strengthened to enable them to use magnesium sulphate appropriately [37].

In evaluating the role of CHW for strengthening child health programmes in Mali, the authors found that provision of continuous training, transport means, adequate supervision and motivation through financial incentives and remuneration are important [38]. In a similar study done in Pakistan to evaluate task sharing with Lady Health Workers for the identification and management of pre-eclampsia, the authors concluded that appropriate training, equipment availability and supervision is a must for successful implementation [39]. In this study too, the need for training and adequate supervision were brought up during the discussions, to be important pre-requisites for the proposed task-sharing. Many studies have reported significant capacity for skill development if the trainees have refresher training in skills to which they were not previously exposed, this being one of the most important factors favouring task-sharing [15].

Recommendations and guidelines have been framed for task- sharing but it has been emphasized that the lower cadre of providers who may be entrusted with new responsibilities will have to fit into the existing health system framework. This is unique to each region, and hence national and local bodies should frame and adopt policies which are relevant to their communities. There must be strong support for these workers based on governance, financing, the supply of medicines and equipment and support from the rest of the formal health system for referral services [8, 40]. Mombo et al. state that task-sharing, if implemented properly, has the potential to play a major role in better access to and more equitable provision of basic health care. However, failure to follow appropriate methods and poor planning and implementation could be counterproductive, with compromised health care delivery [41]. This study found during discussions and interviews that most of the participants favoured task-sharing and emphasised the need for adequate training, supervision and logistical support for the CHW.

## Conclusion

This qualitative study found strong support for task-sharing activities such as home based blood pressure monitoring by community health workers; initiation of emergency treatment and transfer of pregnant women identified as having hypertensive disorders of pregnancy to higher centers by health care providers for further management. The concerns raised were inadequate knowledge, training, experience, supervision and ability of lower cadre health workers to appropriately deliver these services in the community. These concerns could be addressed by community engagement, repeated training to bridge the knowledge gap and active monitoring of the newly assigned tasks by trained personnel. The future implication of this study is to help in implementing a larger trial to evaluate whether these task-sharing can help in reducing mortality and morbidity among pregnant women suffering from HDP and can strengthen existing healthcare infrastructure with constraint human resources.

## Strengths of the study

This study was conducted in collaboration with Indian and multinational researchers who are experienced in developing qualitative protocols and analysis. Their experience has generated robust study design. The local site researchers are familiar with the local Kannada language, which helped participants express their opinions freely and contributed to a better understanding by the facilitators.

## Limitations of the study

Primary health centre staff identified the participants from the local community and this may have resulted in selection bias. The participants who did not access primary care through the formal health care delivery system were unlikely to be contacted for participation. The participants who were never exposed to such FGDs could have been hesitant in expressing their thoughts freely or may have withheld their comments. Even though the researchers were well-trained to facilitate equal inputs from all the participants, group dynamics and cultural barriers may have enhanced or hindered the dialogues by some participants. Non–probabilistic sampling methods limit the ability to generalize results.

## Abbreviation

ANM: Auxiliary nurse-midwives
ASHA: Accredited social health activists
CHW: Community health workers
DGO: Diploma in gynaecology and obstetrics
FGD: Focus group discussion
HDP: Hypertensive disorders of pregnancy
IDI: In-depth interview
MBBS: Batchelor of medicine/bachelor of surgery
MD: Medicine doctor
OBGYN: Obstetrician and gynaecologist
SBA: Skilled birth attendant
WHO: World Health Organisation
WRA: Women of reproductive age
